# Mesenchymal stem cells promote caspase-3 expression of SH-SY5Y neuroblastoma cells via reducing telomerase activity and telomere length

**DOI:** 10.22038/IJBMS.2021.59400.13187

**Published:** 2021-11

**Authors:** Ezzatollah Fathi, Somayeh Vandghanooni, Soheila Montazersaheb, Raheleh Farahzadi

**Affiliations:** 1 Department of Clinical Sciences, Faculty of Veterinary Medicine, University of Tabriz, Tabriz, Iran; 2 Hematology and Oncology Research Center, Tabriz University of Medical Sciences, Tabriz, Iran; 3 Molecular Medicine Research Center, Tabriz University of Medical Sciences, Tabriz, Iran

**Keywords:** Caspase3, Mesenchymal stem cells, Neuroblastoma, Signaling pathways, Telomerase, Telomere

## Abstract

**Objective(s):**

The use of mesenchymal stem cells in malignancies has attracted much attention due to their ability to deliver anticancer agents to tumors, including cytokines, chemokines, etc. This study aimed to investigate the effect of MSCs on the neuroblastoma SH-SY5Y cells through proliferation/apoptosis, senescence assessment, telomere length, and telomerase activity in vitro. BAX and BCL2 were also examined as potential signaling pathways in this process.

**Materials and Methods:**

For this reason, two cell populations (MSCs and SH-SY5Y cells) were co-cultured on trans-well plates for 7 days. In a subsequent step, SH-SY5Y cells were harvested from both control and experimental groups and subjected to flow cytometry, ELISA, real-time PCR, PCR-ELISA TRAP assay, and Western blotting assay for Ki67/Caspase3 investigation, β-Galactosidase assessment, telomere length, and telomerase activity assay. Also, expression of genes and proteins through real-time PCR and Western blotting demonstrated the involvement of the aforementioned signaling pathways in this process.

**Results:**

It was found that MSCs contributed significantly to decrease and increase of Ki-67 and Caspase-3, respectively. Also, MSCs dramatically reduced the length of telomere and telomerase activity and increased the β-Galactosidase activity in a significant manner. In addition, significant increase and decrease were also seen in BAX and BCL2 gene and protein expressions, respectively.

**Conclusion:**

These findings revealed that close interaction between MSCs and neuroblastoma cells causes inhibition of the SH-SY5Y cell proliferation and promotes cell senescence via BAX and caspase-3 cascade pathways.

## Introduction

Neuroblastoma is an extracranial tumor that causes death in childhood (1). Different therapeutic strategies include surgery, radiotherapy, chemotherapy, etc. But the outcome of these methods has definitely not been satisfactory. For this reason, over the past few years, researchers have increasingly focused on stem cell transplantation (2). Regarding the secretion of cytokines, chemokines, and anticancer factors, mesenchymal stem cells (MSCs) are ideally suited for cell-based therapy (3, 4). MSCs are multipotent cells isolated from various sources like amniotic fluid, bone marrow (BM), umbilical cord, adipose tissue and can differentiate into cells of mesodermal lineage (5, 6). MSCs are individually appropriate for the role of the carrier for anticancer therapies. In other words, MSCs are immunologically quiescent because of the low expression of major histocompatibility complex 1 (MHC1) and are able to migrate into the tumor site (7). Some studies indicated the capacity of MSCs homing to tumors that is favored in preclinical models of cancers (8, 9). In general, studies regarding the MSCs’ effects on cancer cells are contradictory. Such that, some studies reported inhibition effects of these cells on tumor growth and others indicated opposite effects (10, 11). MSCs may favor tumor proliferation via stimulation of the metastatic potential and also prevent tumor cell recognition by the immune system. Conversely, MSCs can exert anticancer activities by down-regulating activities through the expression of apoptotic molecules. To date, the effects of MSCs on neuroblastoma growth and the mechanisms of influences on tumor progression via telomere length and telomerase activity are yet to be reported. Therefore, this study reveals the *in vitro* effects of MSCs on the proliferation and apoptosis of the SH-SY5Y cells as neuroblastoma cell lines by investigating the Annexin-V and Caspase-3 assay, telomerase, and β-Galactosidase activity as well as telomere length. The possible involved signaling pathways like BAX as well as BCL2 were also investigated.

## Materials and Methods

**Materials**

All materials related to cell culture as well as the culture polystyrene plates, if not otherwise specified, were obtained from SPL Life Sciences Co., Ltd. (Gyeonggi-do, Korea). 

***Cell culture***

Adipose tissue-derived-MSCs and SH-SY5Y neuroblastoma cells were purchased from Royan Institute and Pasteur Institute of Iran, respectively. MSCs and SH-SY5Y cells were cultured in DMEM low glucose (Gibco, UK) containing 10% FBS (complete culture medium). The medium was replaced twice weekly during the cultivation of the cells. MSCs of passages 3–6 were used throughout the present study (12).

***Characterization of MSCs***

Purchased MSCs were characterized by flow cytometry with antibodies against cell surface markers CD73, CD44, CD31, and CD34. Preparation of cells for flow cytometry method was previously described by Fathi *et al*. (2020) (13). Briefly, nearly 10^6^ MSCs were trypsinized and incubated with 10 µg/10^6^ cells FITC-conjugated antibody CD31 and CD34 and PE-conjugated CD73, and CD44 in washing buffer (PBS supplemented with 3-5% FBS) on ice. In the end, the cells were washed with washing buffer, and the FACS instrument was used to quantify the fluorescence intensity of MSCs (14). 

***Co-culture of MSCs and neuroblastoma cell line (SH-SY5Y)***

MSCs were seeded into 6-well trans-well plates at 10^5 ^cells/well in DMEM complete culture medium. After 24 hr of cells culture, 10^6 ^SH-SY5Y cells/well were added respectively into two groups: control group (culture of SH-SY5Y cells alone) and experimental group (co-cultured SH-SY5Y cells and MSCs). At the end of the co-culture period (7 days), two groups of cells were subjected to total RNA isolation for gene expression, Ki/caspase-3 assessment by flow cytometry, and protein extraction for Western blotting.

***Flow cytometric investigation of apoptosis assay***

To determine cell apoptosis in co-culturing SH-SY5Y cells with MSCs, Annexin-V staining was used. Annexin-V can bind to phosphatidylserine at the surfaces of apoptotic cells as a marker for apoptosis. In this regard, Annexin-V expression was detected with a flow cytometer in FITC and PI channels, exhibiting early and late apoptosis, respectively. For this purpose, the Annexin-V staining detection kit was used to measure apoptosis in 10^6^ cells from both the control and experimental groups. according to manufacturer’s directions (Ref No: 11-8005-74, e-bioscience). FlowJo software ver. X.0.7 was then used to analyze the percentages of viable and apoptotic cells (15, 16). 

***Ki-67/caspase-3 investigation***

Ki-67/caspase-3 assay was performed in control and experimental groups. In this regard, the cells were incubated with 0.2% Triton X-100. Next, the cells were stained with Ki-67 antibody for 30 min. Moreover, for the caspase-3 assay, cells were fixed using FCM fixation buffer and permeabilized by FCM permeabilization buffer. Washed cells were stained using PE-conjugated anti-caspase. The flow cytometry data were analyzed with FlowJo software ver. X.0.7 (17).

***Absolute telomere length (aTL) measurement and telomerase activity assay***

For measuring the telomere length, the total DNA was isolated (18). The aTL measurement was done by real-time PCR as previously reported by Fathi *et al*. (2020) (19). Also, the primers used in this method were previously designed by O’Callaghan and Fenech (2011) (Table 1) (20). The telomerase activity of SH-SY5Y cells was measured by PCR-ELISA TRAP assay. 

***Senescence-associated β-galactosidase assay by ELISA***

Cells (10^6^ cells/wells) from both groups (control and experimental) were lysed and β-Galactosidase activity was assessed using the human β-Galactosidase enzyme-linked immunosorbent assay (ELISA) Kit (11539426001, Roche, UK) according to the manufacturer’s guidelines (21).

***Real time-PCR***

After the end of treatment time (7 days), 10^6^ SH-SY5Y cells/well from both groups of cells were collected, total RNA from the cells was isolated and cDNA was synthesized using a YTA kit (Yekta Tajhiz Azma, IRAN) (22). The mRNA expressions of target genes in this experiment included *BAX, BCL2, *and *β-actin*. The primer sequences used for real-time PCR are listed in Table 2.

***Western blotting assay***

Western blotting was used to determine whether BAX/BCL2 signaling pathways are involved. For this examination, proteins were extracted from both control and experimental groups of SH-SY5Y cells and were electrophoresed using 12% polyacrylamide, and finally transferred onto PVDF membranes. Following that, the membrane was exposed overnight to primary antibodies for BAX and BCL2 (1:500, Santa Cruz Biotechnology, CA). Afterward, the membranes were incubated at 25 °C for 60 min with a secondary antibody (1:5000 Santa Cruz). Next, X-ray films were used to detect the presence of protein bands. Protein bands were quantified and represented as the ratio of target protein/β-actin and the values were then graphed (23, 24). 

***Statistical analysis***

Values were considered statistically significant at *P*<0.05 using Graph Pad Prism version 6.01 using t-test and Two-way ANOVA followed by Dennett’s *post hoc* test. Flow cytometry and real-time PCR were analyzed by FlowJo and REST 2009 software packages, respectively. 

## Results

***Characterization of MSCs***

**Figure 1:**
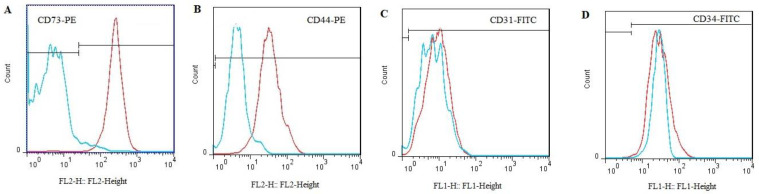
Expression of adipose tissue derived-MSC cell surface markers by flow cytometry. During this experiment, each antibody was examined separately, and isotope controls served as negative controls; adipose-derived MSCs were positively characterized by CD73 (94%) and CD44 (80%), while CD31 (0.2%) and CD34 (0.04%) were negative. Isotype controls of CD31 and CD34 are depicted as blue diagrams

After de-freezing and culturing the MSCs, cell surface marker characterization was performed according to Farahzadi *et al*. (2016) (25). As shown in Figure 1 (A-D), MSCs expressed the cell surface markers CD73 (94%) and CD44 (80%). Also, the cell surface markers related to hematopoietic cells CD31 (0.2%) and CD34 (0.04%) are not expressed in these cells. 

***Cell apoptosis investigation***

**Figure 2:**
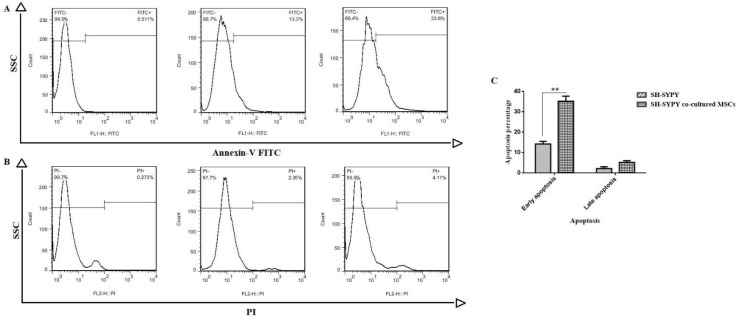
Co-culturing MSCs and SH-SY5Y cells was analyzed with Annexin-FITC and PI flow cytometric method. The analysis was conducted on two distinct populations of cells marked as: negative cells indicating live cell population, (A) Annexin-V positive cells, showing early apoptosis; (B) PI-positive cells, showing late apoptosis

As previously reported by investigators, early and late apoptotic cells are Annexin V positive (Annexin^+^) and PI (PI^+^), respectively. Figure 2 shows the diagrams of Annexin V and PI stained SH-SY5Y cells after the co-culture period. In more detail, early apoptosis (Annexin^+^) was about 33.6% for SH-SY5Y cells exposed to MSCs, which was 2.52 times higher than that of the control group (13.3%). While it was only 4.11% for the cells in the late apoptotic stage (PI+) (Figure 2).

***Proliferation/apoptosis investigation by Ki-67/caspase-3 assay***

**Figure 3:**
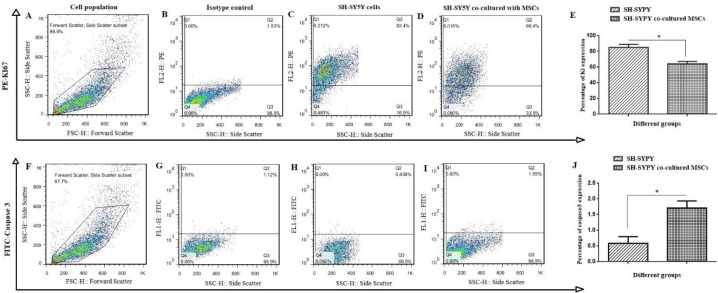
Proliferation assay of SH-SY5Y cells and SH-SY5Y cells co-cultured with MSCs. Flow cytometry was used to analyze the harvested cells for Ki-67 (A-E) and caspase-3 expression assay (F-J). In this Figure, A and F represent selected cell populations, B and G are isotype controls, C and H are SH-SY5Y cells, finally, D and I show co-cultured SH-SY5Y with MSCs cells. Data is represented as mean ± SD of independent experiments; (*P<0.05, n=3)

The effect of MSCs on cell proliferation of SH-SY5Y cells was investigated by Ki-67 expression (Figures 3A-E). The percentage of Ki-67 expression in the co-culture group (66.4%) was lower than that in the control group (82.4%) (Figures 3A-E) (*P*<0.05). Also, Figures 3F-J showed that caspase-3 levels in the co-culture group were increased about 3.56-fold compared with the control group (Figures 3F-J) (*P*<0.05).

***Investigation of aTL and telomerase activity***

**Figure 4:**
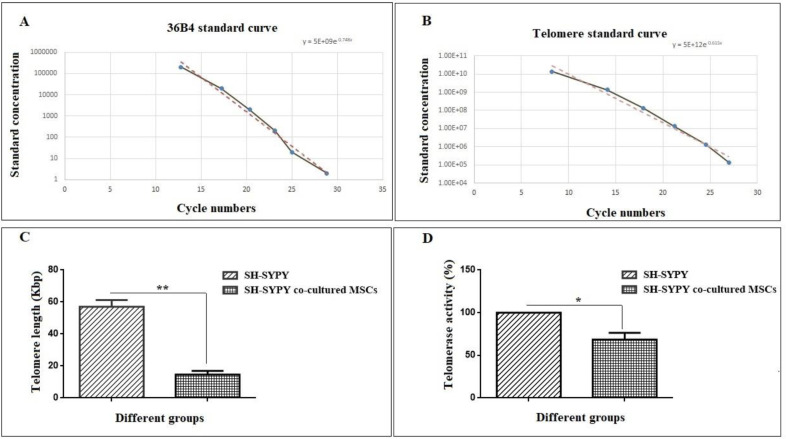
aTL measurement of SH-SY5Y cells following co-culture with MSCs. (A) shows the standard curve for calculating genome copies using the 36B4 copy number, and (B) shows the standard curve for calculating the length of telomere sequence per reaction tube (the X-axis represents the number of the cycle and the Y-axis shows the standard’s concentration). (C) For both control and experimental groups, real-time PCR analysis was performed in triplicate to evaluate telomere length. The data were analyzed as kb/reaction and the genome copies/reaction for the telomere and the SCG. Values are mean ± SD from independent experiments (**P<0.01, n=3); (D) Relative telomerase activity measurement of SH-SY5Y cells following co-culture with MSCs. From each group of cells (control and experimental), 1×10^6 ^cells were collected per well. In the following step, protein was extracted and PCR-ELISA TRAP assay was carried out in triplicate using the method detailed in the materials and methods section. Values are mean ± SD from independent experiments ;(*P<0.05, n=3)

As shown in Figure 4C, aTL was significantly decreased (14.60 Kbp) compared with the control group (57 Kbp) (*P*<0.01). Also, the telomerase activity was decreased by 68.5% in the experimental group compared with the control group (Figure 4D) (*P*<0.05). 

***β*** ***-*** ***Galactosidase*** ***activity for cell senescence investigation***

**Figure 5:**
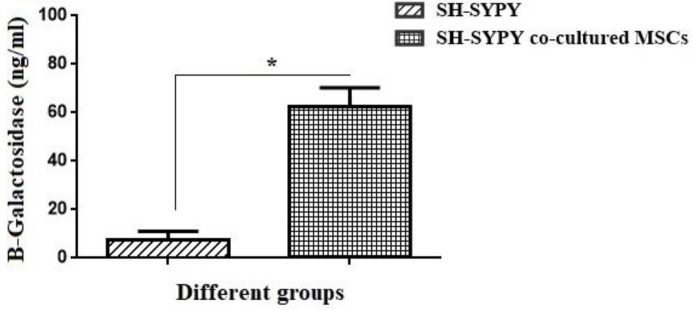
β-GAL activity assay of SH-SY5Y cells and SH-SY5Y cells co-cultured with MSCs

As shown in Figure 5, the concentration of β-Galactosidase in the experimental group was 8.33 folds higher than that of the control group (*P*<0.05) (Figure 5).

***MSCs change mRNA and protein expression of BAX and BCL2 in the SH-SY5Y cell line***

**Figure 6:**
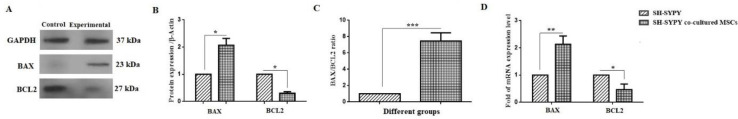
Effect of MSCs on apoptosis-related gene and protein expression. (A and B) BAX and BCL-2 protein expression (C) BAX/BCL2 ratio, (D) Relative level of mRNA expression of Bax and BCL2. Data are expressed as mean ± SD from three independent experiments ;(*P<0.05, **P<0.01, ***P<0.001, n=3)

As shown in Figures 6A and B, the protein expression levels of BAX and BCL2 were significantly increased and decreased, respectively (*P*<0.05). In addition, the apoptotic BAX/BCL-2 ratio was increased by 7.7 folds in the co-cultured SH-SY5Y cell line (Figure 6C) (*P*<0.001). A significant increase and decrease in mRNA expression levels of BAX and BCL2, respectively, were also seen (Figure 6D) (*P*<0.05, *P*<0.01).

**Table 1:** Oligomers and characterizations used for absolute telomere length measurement

**Oligomer name**		**Oligomer sequence (5'-3')**	**Molecular weight** **(MW)**	**Other calculations**		**Purification method**
To draw a standard curve	Telomere standard	(TTAGGG)14	26667.2.	**Weight (g)**	2.6667×10^4^/6.02×10^23^= 0.44×10^-19^	HPLC
				**Number molecules of oligomer in TEL STD A**	60×10^-12^/0.44× 10^-19^=1.36×10^9^	
				**Amount of telomere sequence in TEL STD A (kbp)**	1.36 ×10^9^×84= 1.18×10^8^	
	36B4 standard	5'CAGCAAGTGGGAAGGTGTAATCCGTCTCCACAGACAAGGCCAGGACTCGTTTGTACCCGTTGATGATAGAATGGG 3'	23268.1	**Weight (g)**	2.32681×10^4^/6.02×10^23^= 0.38×10^-19^	
				**Number copies ** **of 36B4**	200×10^-12^/0.44× 10^-19^= 5.26×10^9^	
To calculate telomere length	Telo	Fwd: CGGTTTGTTTGGGTTTGGGTTTGGGTTTGGGTTTGGGTT Rev: GGCTTGCCTTACCCTTACCCTTACCCTTACCCTTACCCT				
	36B4	Fwd: CAGCAAGTGGGAAGGTGTAATCC Rev: CCCATTCTATCATCAACGGGTACAA				

**Table 2:** Primer sequences used for real time-real time-polymerase chain reaction

**No.**	**Gene**	**Primer pair sequence (5'-3')**	**Product length (bp)**
NM_138761.4	BAX	TGCCAGCAAACTGGTGCTCA GCACTCCCGCCACAAAGATG	194
NM_000633.2	BCL2	TCTGTGGATGACTGAGTACCTGAAC AGAGACAGCCAGGAGAAATCAAA	129
NM_017008.4	GAPDH	ATGACTCTA CCCACGGCAAG CTGGAGATGGTGATGGGTT	88

The quantification analysis of apoptosis was depicted in part C. Date are expressed as mean ± SD from three independent experiments (**P<0.01, n=3)

compared with the control group, the experimental group displayed a higher level of β-galactosidase activity. Values are mean ± SD from independent experiments ;(*P<0.05, n=3)

## Discussion

In cancer treatment, stem cells can act as a promising delivery platform by homing to and delivering therapeutics into tumor foci (26). Studies regarding the role of MSCs in cancer cells are contradictory. Some of these are associated with their promotional effects, while others address their inhibitory effects (8, 27-28). Only limited researches have been conducted on the effect of MSCs on neuroblastoma cells/tumors. A study by (2008) found that BM-derived MSCs are capable of differentiating into Schwann cells in neuroblastoma tumors both *in vitro* and *in vivo*. Based on these findings, Schwannian stroma in neuroblastoma tumors may have originated from non-neoplastic cells rather than neuroblastoma cells (29). In another clinical study (2010), the efficiency and safety of infusing autologous BM-derived-MSCs for treating neuroblastoma patients were reported (30). They demonstrated that MSCs can be used as tools to deliver oncolytic adenoviruses to neuroblastoma tumors with beneficial antitumor effects and low toxicity (30). Another study (2012) indicated that although the intratumoral injection of MSCs in neuroblastoma nodules inhibited tumor growth, systemically, it did not home to neuroblastoma tumors and was not suitable for selective antitumor drugs delivery at the neuroblastoma site (8). However, the precise cellular and molecular mechanisms of MSCs’ effects on cancers and tumor progression are yet to be elucidated. Our study presents evidence for the effects of MSCs on SH-SY5Y neuroblastoma cells by investigating Annexin-V and Caspase-3 assay, telomere length, telomerase, and β-Galactosidase activity assessment through BAX and BCL2 signaling pathways. To explore the hypothesis of this study, SH-SY5Y cells were co-cultivated with MSCs in a trans-well system for 7 days. Both the control and experimental groups of SH-SY5Y cells were collected at the end of co-culture time, and flow cytometry, real-time PCR, PCR-ELISA TRAP assay, Western blot, and ELISA were performed. Based on the results of this study, the experimental group (SH-SY5Y co-cultured with MSCs) exhibited a dramatic decline in telomere length and telomerase activity which was accompanied by a remarkable increment in the β-Galactosidase activity by more than 3.9, 1.45, and 8.33 fold, respectively, in comparison with the control group consisting of SH-SY5Y cells alone. Due to high telomerase activity in cancer, its inhibition is a great interest and strategy in cancer treatment. Despite numerous studies, the underlying molecular and cellular mechanisms have yet to be fully elucidated. 

Previous investigations have reported that telomere length and telomerase activity were considered as useful diagnostic and prognostic markers in some neoplasms such as neuroblastoma (31). So that a high correlation was demonstrated between telomere length and malignancies in brain tumors including meningioma and glioblastomas (32). In one study (2004), it was shown that shorter and elongated telomeres were accompanied by favorable and poor prognoses, respectively (33). With these explanations, the results of our study regarding the telomere length are consistent with the previous findings. Based on the results of previous studies regarding the involved mechanisms in telomere length change and telomerase activity, it was theorized that shortening of telomere length and telomerase activity may be strongly influenced by MSCs-secreted cytokines released (34). In this regard, a number of factors may have a contributory role, including IL-6, IL-8, and TGF-β.

Aside from measuring telomere length, we assessed telomerase and β-Galactosidase activity, as well as Annexin-V staining, Ki-67/Caspase-3 assay, BAX, and BCL2 levels. Clear identification of the mechanism of cell damage/death caused by MSCs is essential for assessing the biological response to cell therapy.

In accordance with the Annexin-V results, it was well-identified that the early apoptotic cells showed a significant increment, with 13.3 apoptotic cells in the control group reaching 33.6% in the experiment group. Additionally, the late apoptotic cell level clearly increased from 2.35 to 4.11% after 7 days of co-culture.

## Conclusion

We provided evidence that MSCs decreased telomerase activity and telomere length of SH-SY5Y cells, which was associated with apoptosis induction and an increase in the expression level of caspase-3. Furthermore, these effects result in increased and decreased expression of the BAX and BCL2, respectively, which are critical components of apoptotic signaling pathways. Finally, a cytokine antibody array method is suggested to investigate the mechanisms by which cytokines are secreted from MSCs, as well as the types of secreted cytokines and growth factors.

## Authors’ Contributions

EF as the main colleague contributed to performing experiments, data analysis, and manuscript writing; SV and SM as main colleagues were involved in manuscript writing; RF as the executive of the project was involved in conception and design, manuscript writing, and supervised the manuscript preparation. 

## Etical Approval

Ethical consent was approved by an ethics committee at Tabriz University of Medical Sciences, Tabriz, Iran (Ethic Code No: IR.TBZMED.VCR.REC.1400.231).

## Conflicts of Interest

The author(s) declare that there are no conflicts of interest.
